# Supplementary material for: Heterosis in crosses between remnant populations of the rare prairie forb Silene regia: Implications for restoration genetics
Source: Am J Bot. 2026 Oct 14;113(10):e70278. doi: 10.1002/ajb2.70278 (PMC13716920; doi:10.1002/ajb2.70278)
Supplement: Supplementary file 2 [file AJB2-113-e70278-s002.docx]

**Appendix S6.** Estimates of overall heterosis (outbreeding depression for negative values) for fitness components and composite fitness estimates, averaged over all three maternal populations (Fou, Tip, and Ver) of *Silene regia* from western Indiana. Means for fitness components and composite estimates of fitness for each cross type, averaged over population cross-type means (see Appendix S3), are also presented. Adult fitness in the greenhouse was estimated by the total number of flowers per plant transplanted, and adult fitness in the field was estimated by the total number of fruits produced over the entire experiment per plant transplanted. Cumulative fitness was calculated separately for the greenhouse and field experiments as the product of four estimates: (1) seed number per fruit, (2) proportion germination, (3) proportion juvenile survival, and (4) adult fitness. Significant contrasts corresponding to estimates of heterosis are indicated where appropriate; **P* < 0.05. Significance testing of cumulative fitness was not possible (see Materials and methods).

| Fitness component | Overall heterosis (%) | BET_pool_ Mean | WIN_pool_ Mean |
| --- | --- | --- | --- |
| Early |  |  |  |
| Seed number per fruit | 10 | 40.9 | 37.0 |
| Germination (%) | 4 | 60.1 | 57.7 |
| Juvenile survival (%) | 6* | 81.1 | 76.8 |
| Greenhouse |  |  |  |
| Survival and reproduction (%) | -9 | 79.4 | 87.5 |
| Fecundity | 19* | 47.3 | 39.8 |
| Flowers per seedling planted | 8 | 38.0 | 35.2 |
| Field |  |  |  |
| Survival and reproduction (%) | 10 | 39.4 | 35.8 |
| Fecundity | 38* | 15.0 | 10.8 |
| Fruits per seedling planted | 45* | 6.3 | 4.3 |
| Cumulative |  |  |  |
| Greenhouse | 35 | 775.6 | 575.5 |
| Field | 112 | 139.0 | 65.4 |
