# Supplementary material for: Heterosis in crosses between remnant populations of the rare prairie forb Silene regia: Implications for restoration genetics
Source: Am J Bot. 2026 Oct 14;113(10):e70278. doi: 10.1002/ajb2.70278 (PMC13716920; doi:10.1002/ajb2.70278)
Supplement: Supplementary file 3 [file AJB2-113-e70278-s003.docx]

**Appendix S4.** Mean fitness components following hand pollinations for each of the three maternal populations of *Silene regia* from western Indiana (Fou, Tip, and Ver) and cross type (WIN = within-population cross, BET = between-population cross) separately by the two paternal populations for between-population crosses. Means and standard errors were calculated from block means, except for seed number per fruit which are the raw means and SE. Sample sizes are given in Appendix S3.
