# Supplementary material for: Heterosis in crosses between remnant populations of the rare prairie forb Silene regia: Implications for restoration genetics
Source: Am J Bot. 2026 Oct 14;113(10):e70278. doi: 10.1002/ajb2.70278 (PMC13716920; doi:10.1002/ajb2.70278)
Supplement: Supplementary file 4 [file AJB2-113-e70278-s004.docx]

**Appendix S7.** Estimates of heterosis (outbreeding depression for negative values) for each of the three maternal populations (Fou, Tip, and Ver) of *Silene regia* from western Indiana for fitness components and composite fitness estimates. Adult fitness in the greenhouse is estimated by the total number of flowers per seedling (sdl.) transplanted, and adult fitness in the field is estimated by the total number of fruits produced over the entire experiment per plant transplanted. Both measures of adult fitness incorporate zero for plants that failed to survive and reproduce (rep.). Cumulative fitness was calculated separately for the greenhouse and field experiments as the product of four estimates: (1) seed number per fruit, (2) proportion germination, (3) proportion juvenile survival (surv.), and (4) adult fitness. The first three columns are values for averaging over all three populations for the within-population cross approach used for the main results. The next three columns give values for the comparison of within- versus between-population crosses separately by maternal population. The remaining columns give the between-population values relative to the average of the within-population values for every possible combination of two populations in each direction (maternal × paternal). Within each set of columns significant contrasts are indicated; †0.05 < *P* < 0.1, **P* < 0.05, ***P* < 0.01, ****P* < 0.001. Significance testing of cumulative fitness was not possible (see Materials and Methods).

| Fitness component | **Fou_pool_** | **Tip_pool_** | **Ver_pool_** | Fou | Tip | Ver | Fou x Tip | Fou x Ver | Tip x Fou | Tip x Ver | Ver x Fou | Ver x Tip |
| --- | --- | --- | --- | --- | --- | --- | --- | --- | --- | --- | --- | --- |
| Early |  |  |  |  |  |  |  |  |  |  |  |  |
| Seed no. / fruit | **41** | **-14** | **4** | 9 | 3 | 19 | 35 | 28 | -20 | 1 | -9 | 28 |
| Germination (%) | **23**** | **-7** | **-3** | 17† | -14 | 12 | 12 | 33** | -13 | -4 | -11 | 16 |
| Juvenile surv. (%) | **13***** | **3** | **1** | 19******* | 1 | -2 | 14******* | 16******* | 1 | 1 | -2 | -1 |
| Greenhouse |  |  |  |  |  |  |  |  |  |  |  |  |
| Surv. and rep. (%) | **-13** | **-11** | **-3** | -12 | -15 | 0 | -12 | -9 | -14 | -8 | 2 | -5 |
| Fecundity | **12** | **8** | **36**** | 15 | 4 | 37* | 7 | 9 | -2 | 19 | 31***** | 34** |
| Flowers/sdl. planted | **-3** | **-6** | **32** | 1 | -12 | 36 | -6 | -1 | -17 | 9 | 31 | 29 |
| Field |  |  |  |  |  |  |  |  |  |  |  |  |
| Surv. and rep. (%) | **23†** | **-5** | **11** | 89******* | 47 | -34******* | 55***** | 23† | 29 | -9 | -13 | 4 |
| Fecundity | **55**** | **29** | **30** | 37† | 69† | 18 | 39 | 56** | 22 | 46***** | 31 | 30 |
| Fruits/sdl. planted | **78***** | **20** | **36** | 157******* | 160***** | -26 | 111***** | 85******* | 61 | 27 | 22 | 2 |
|  |  |  |  |  |  |  |  |  |  |  |  |  |
| Cumulative |  |  |  |  |  |  |  |  |  |  |  |  |
| Greenhouse | **92** | **-23** | **35** | 52 | -21 | 78 | 61 | 95 | -42 | 8 | 5 | 90 |
| Field | **281** | **6** | **50** | 289 | 131 | -4 | 262 | 265 | 14 | 26 | -2 | 50 |
