# Supplementary material for: Heterosis in crosses between remnant populations of the rare prairie forb Silene regia: Implications for restoration genetics
Source: Am J Bot. 2026 Oct 14;113(10):e70278. doi: 10.1002/ajb2.70278 (PMC13716920; doi:10.1002/ajb2.70278)
Supplement: Supplementary file 5 [file AJB2-113-e70278-s005.docx]

**Appendix S1.** ANOVA results for early fitness components of offspring derived from hand pollinations within and between three populations (Fou, Tip, and Ver) of *Silene regia* from western Indiana. Fixed effects include the effects of maternal stratification treatment (Mat. strat. trt.), maternal and paternal populations and their interaction, and block (where appropriate, otherwise n/a). Models for both seed number per fruit and percentage germination (per cell) employed normal error distributions. The model for juvenile survival employed a binomial error distribution. Contrasts between cross types are only reported for models with *P* <0.1 for either the interaction between maternal population and paternal population, or both main effects of maternal and paternal populations; otherwise, these contrasts are n/a. For the contrasts, WIN = within-population cross, BET = between-population cross, and the subscript pool indicates that the contrast involves all individuals of a given cross type (averaged over all three populations), and contrasts that start with a population code followed by a colon test for heterosis or outbreeding depression separately by maternal population. Model results were similar to models without incorporating effects of maternal stratification treatment (compare with Table 1), so we proceed with the simpler model throughout the results.

|  | Seed number per fruit  (Denom. DF = 227) | | Germination  (Denom. DF = 344) | | Juvenile Survival | |
| --- | --- | --- | --- | --- | --- | --- |
| Model terms | *F* | *P* | *F* | *P* | $\chi$*^2^* | *P* |
| Maternal population | 13.1 | **<0.001** | 6.4 | **0.002** | 5.3 | 0.070 |
| Paternal population | 1.0 | 0.366 | 2.7 | 0.071 | 6.3 | **0.044** |
| Mat. pop. * Pat. pop. | 0.3 | 0.877 | 1.1 | 0.345 | 14.6 | **0.006** |
| Mat. Strat. Trt. | 13.5 | **<0.001** | 2.0 | 0.132 | 0.4 | 0.804 |
| Block | n/a | n/a | 4.5 | **0.002** | 64.9 | **<0.001** |
|  |  |  |  |  |  |  |
| Contrast | *t* | *P* | *t* | *P* | $\chi$*^2^* | *P* |
| BET_pool_ vs. WIN_pool_ | n/a | n/a | 0.3 | 0.758 | 6.0 | **0.015** |
| Fou: BET vs. WIN_pool_ | n/a | n/a | 2.8 | **0.005** | 21.5 | **<0.001** |
| Tip: BET vs. WIN_pool_ | n/a | n/a | -1.0 | 0.305 | 0.5 | 0.458 |
| Ver: BET vs. WIN_pool_ | n/a | n/a | -0.6 | 0.520 | 0.3 | 0.577 |
| Fou: BET vs. WIN | n/a | n/a | 1.9 | 0.064 | 30.9 | **<0.001** |
| Tip: BET vs. WIN | n/a | n/a | -1.5 | 0.142 | 0.0 | - |
| Ver: BET vs. WIN | n/a | n/a | 0.6 | 0.525 | 0.0 | - |
