# Supplementary material for: Heterosis in crosses between remnant populations of the rare prairie forb Silene regia: Implications for restoration genetics
Source: Am J Bot. 2026 Oct 14;113(10):e70278. doi: 10.1002/ajb2.70278 (PMC13716920; doi:10.1002/ajb2.70278)
Supplement: Supplementary file 6 [file AJB2-113-e70278-s006.docx]

**Appendix S2. Schematic of crossing design, example comparisons of cross types, and list of fitness components and composite estimates of fitness in each experiment. The crossing design within (gray) and between (black) three populations of *Silene regia* from western Indiana (Fou, Tip, and Ver) is shown in panel A. For between population crosses, the arrow points from the paternal population to the maternal population. Panels B and C show the two different approaches for estimating heterosis using Fou as an example maternal population. Panel** B represents the simplistic approach where only the within-population crosses for a given maternal population are used. In panel C, heterosis is measured as the increase in fitness of the mean of the between-population crosses (averaged over the two paternal populations) relative to the mean of the within-population (averaged over all three populations) crosses (WIN_pool_) to account for additive difference in mean fitness of within population crosses between the parents**. Panel D lists the fitness components measured in each experiment and indicates how cumulative fitness was estimated for the greenhouse and field experiments.** Cumulative fitness was calculated separately for the greenhouse and field experiments as the product of four estimates: (1) seed number per fruit, (2) proportion germination, (3) proportion juvenile survival, and (4) adult fitness.
