# Supplementary material for: Heterosis in crosses between remnant populations of the rare prairie forb Silene regia: Implications for restoration genetics
Source: Am J Bot. 2026 Oct 14;113(10):e70278. doi: 10.1002/ajb2.70278 (PMC13716920; doi:10.1002/ajb2.70278)
Supplement: Supplementary file 8 [file AJB2-113-e70278-s008.docx]

**Appendix S9.** Mean fitness components after hand pollinations for each of the three maternal populations of *Silene regia* from western Indiana (Fou, Tip, and Ver) and cross type (WIN = within-population cross, BET = between-population cross) for the combined probability of survival and reproduction, fecundity and fruits per seedling planted in the field experiment. Means and standard errors were calculated from the means within each of the six blocks. Within a population, significant contrasts of BET vs. WIN are indicated where appropriate; †0.05 < *P* < 0.1, **P* < 0.05, ****P* < 0.001 (see Table 3).
