# Supplementary material for: Heterosis in crosses between remnant populations of the rare prairie forb Silene regia: Implications for restoration genetics
Source: Am J Bot. 2026 Oct 14;113(10):e70278. doi: 10.1002/ajb2.70278 (PMC13716920; doi:10.1002/ajb2.70278)
Supplement: Supplementary file 9 [file AJB2-113-e70278-s009.docx]

**Appendix S3.** Sample sizes and mean values (averaged over flats/blocks where appropriate) for fitness components of offspring derived from hand pollinations within and between three populations (Fou, Tip, and Ver) of *Silene regia* from western Indiana. For seed number per fruit, sample size indicates the number of fruits. For germination, mean is the percentage germination per cell (usually starting with 10 seeds) averaged over five flats, and sample size represents the total number of cells over all flats. For juvenile survival (surv.), the mean percent survival over the means of the 30 flats is given, and sample size represents the total number of seedlings over all flats. For the greenhouse experiment, sample sizes are the total number of plants at the start of the experiment over all 48 flats, and means are the mean over flat means. For the field experiment, sample sizes are the total number of plants at the start of the experiment over all six blocks, and means are the mean over block means. For fecundity in both experiments, sample sizes are the number of plants that reproduced. Adult fitness in the greenhouse is estimated by the total number of flowers per plant transplanted, and adult fitness in the field is estimated by the total number of fruits produced over the entire experiment per plant transplanted. Cumulative fitness was calculated separately for the greenhouse and field experiments as the product of four estimates: (1) seed number per fruit, (2) proportion germination, (3) proportion juvenile survival, and (4) adult fitness. No sample size (n/a) is given for cumulative fitness because it is the product of multiple fitness components.

|  | Fou | | | | Tip | | | | Ver | | | |
| --- | --- | --- | --- | --- | --- | --- | --- | --- | --- | --- | --- | --- |
|  | BET | | WIN | | BET | | WIN | | BET | | WIN | |
| Fitness component | *N* | Mean | *N* | Mean | *N* | Mean | *N* | Mean | *N* | Mean | *N* | Mean |
| Early |  |  |  |  |  |  |  |  |  |  |  |  |
| Seed number per fruit | 71 | 52.3 | 39 | 47.8 | 45 | 31.7 | 17 | 30.9 | 47 | 38.6 | 19 | 32.4 |
| Germination (%) | 120 | 71.2 | 60 | 61.0 | 60 | 53.4 | 30 | 62.2 | 59 | 55.8 | 30 | 49.9 |
| Juvenile surv. (%) | 723 | 86.9 | 337 | 73.3 | 307 | 78.9 | 170 | 78.1 | 291 | 77.5 | 149 | 79.0 |
| Greenhouse |  |  |  |  |  |  |  |  |  |  |  |  |
| Surv. and reproduction (%) | 118 | 76.0 | 59 | 86.2 | 59 | 77.5 | 30 | 91.3 | 59 | 84.7 | 29 | 85.1 |
| Fecundity | 112 | 44.7 | 55 | 38.9 | 53 | 42.9 | 28 | 41.1 | 55 | 54.3 | 26 | 39.5 |
| Flowers per seedling planted | 118 | 34.2 | 59 | 34.0 | 59 | 33.1 | 30 | 37.5 | 59 | 46.6 | 29 | 34.2 |
| Field |  |  |  |  |  |  |  |  |  |  |  |  |
| Surv. and reproduction (%) | 239 | 44.0 | 120 | 23.3 | 120 | 34.2 | 60 | 23.3 | 118 | 39.9 | 59 | 60.9 |
| Fecundity | 105 | 16.8 | 28 | 12.3 | 41 | 14.0 | 14 | 8.3 | 47 | 14.1 | 36 | 11.9 |
| Fruits per seedling planted | 239 | 7.7 | 120 | 3.0 | 120 | 5.2 | 60 | 2.0 | 118 | 5.9 | 59 | 8.0 |
| Cumulative |  |  |  |  |  |  |  |  |  |  |  |  |
| Greenhouse | n/a | 1106.7 | n/a | 726.7 | n/a | 442.1 | n/a | 562.9 | n/a | 777.9 | n/a | 436.8 |
| Field | n/a | 249.2 | n/a | 64.1 | n/a | 69.5 | n/a | 30.0 | n/a | 98.5 | n/a | 102.2 |
